# Supplementary material for: The MEK1/2 Inhibitor ATR-002 (Zapnometinib) Synergistically Potentiates the Antiviral Effect of Direct-Acting Anti-SARS-CoV-2 Drugs
Source: Pharmaceutics. 2022 Aug 25;14(9):1776. doi: 10.3390/pharmaceutics14091776 (PMC9506552; doi:10.3390/pharmaceutics14091776)
Supplement: Supplementary file 1 [file pharmaceutics-14-01776-s001.zip › pharmaceutics-1840327-supplementary.pdf]

pharmaceutics

MDPI

The MEK1/2 Inhibitor ATR-002 (Zapnometinib) Synergistically Potentiates the Antiviral Effect of Direct-Acting Anti-SARS-CoV-2 Drugs

André Schreiber, Benjamin Ambrosy, Oliver Planz, Sebastian Schloer, Ursula Rescher and Stephan Ludwig

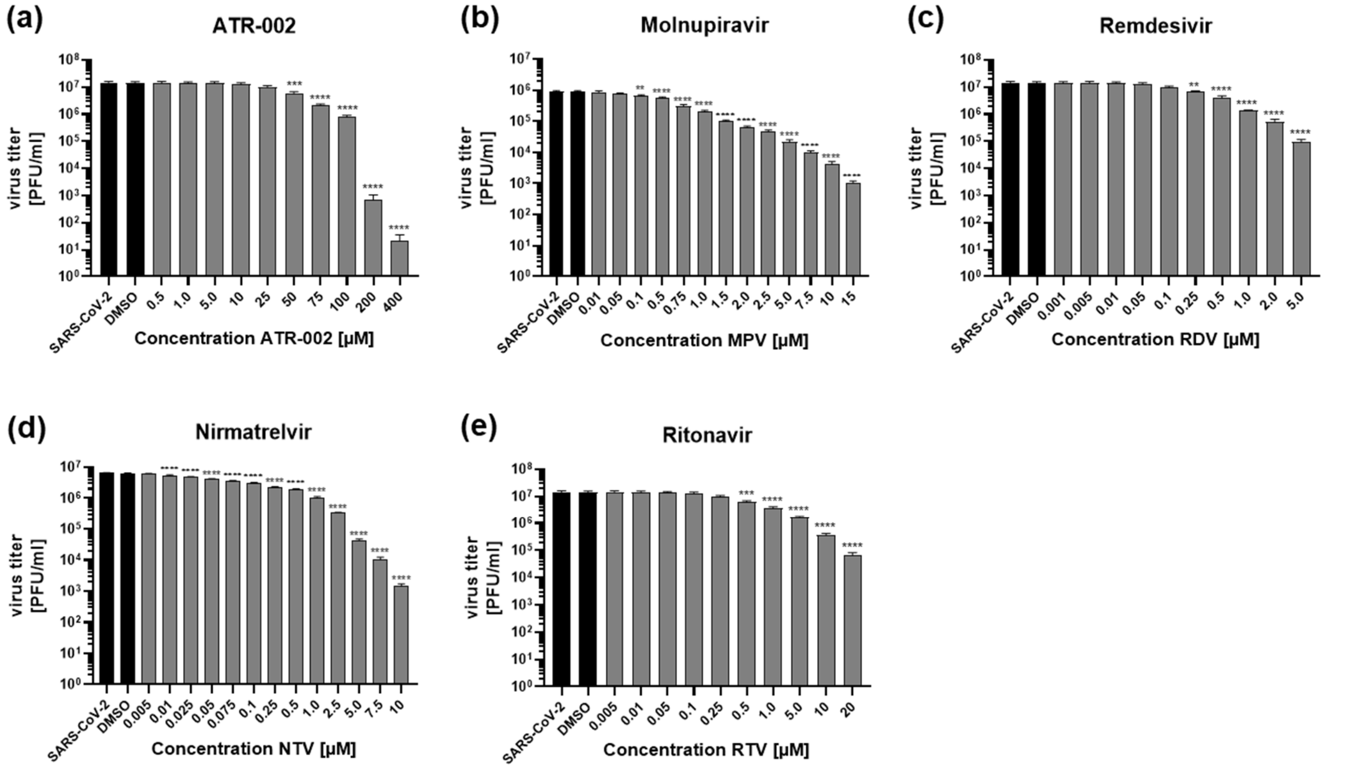

Figure S1. Drug related inhibitory effect on the SARS-CoV-2 replication. ***Related to Figure 1.***

SARS-CoV-2 (MOI: 0.01) was used to infect Calu-3 cells. Drug treatment was initiated 1 h.p.i. Untreated (SARS-CoV-2) and DMSO-treated cells served as negative controls. Viral titers were analyzed 48 h.p.i.

Shown are viral titers in PFU/mL. Data represent means ± SEM of five independent experiments, each performed in triplicates. Significance was calculated by one-way ANOVA in combination with a Dunnett´s multiple comparisons test using DMSO as reference (** $p \leq 0.0021$; *** $p \leq 0.0002$; **** $p \leq 0.0001$).

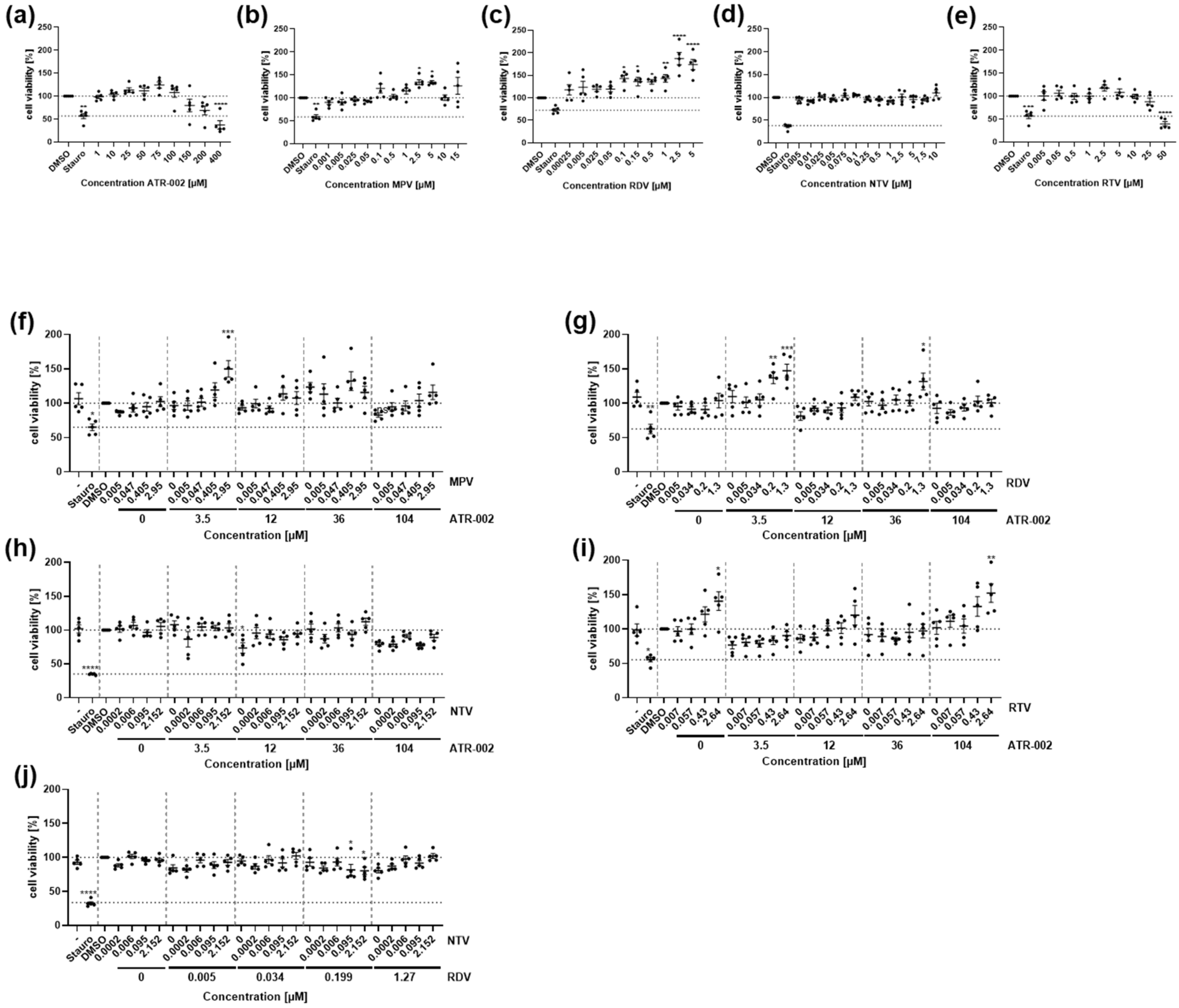

Figure S2. Determination of the cell viability after single and combinational drug treatment.

Calu-3 cells were single treated (**a**–**e**) or double treated (**f**–**j**) with the indicated drug concentrations and combinations. 48 h post-treatment cell viability was evaluated using a MTT-assay. DMSO was used as negative control, Staurosporine (Stauro; 1 µM) as positive control. DMSO and Staurosporine levels are indicated by dashed lines. Shown are means ± SEM of five independent experiments, each performed in triplicates. Significance was calculated using an one-way ANOVA in combination with a Dunnett´s multiple comparisons test with DMSO as reference (* $p \leq 0.0332$; ** $p \leq 0.0021$; *** $p \leq 0.0002$; **** $p \leq 0.0001$).

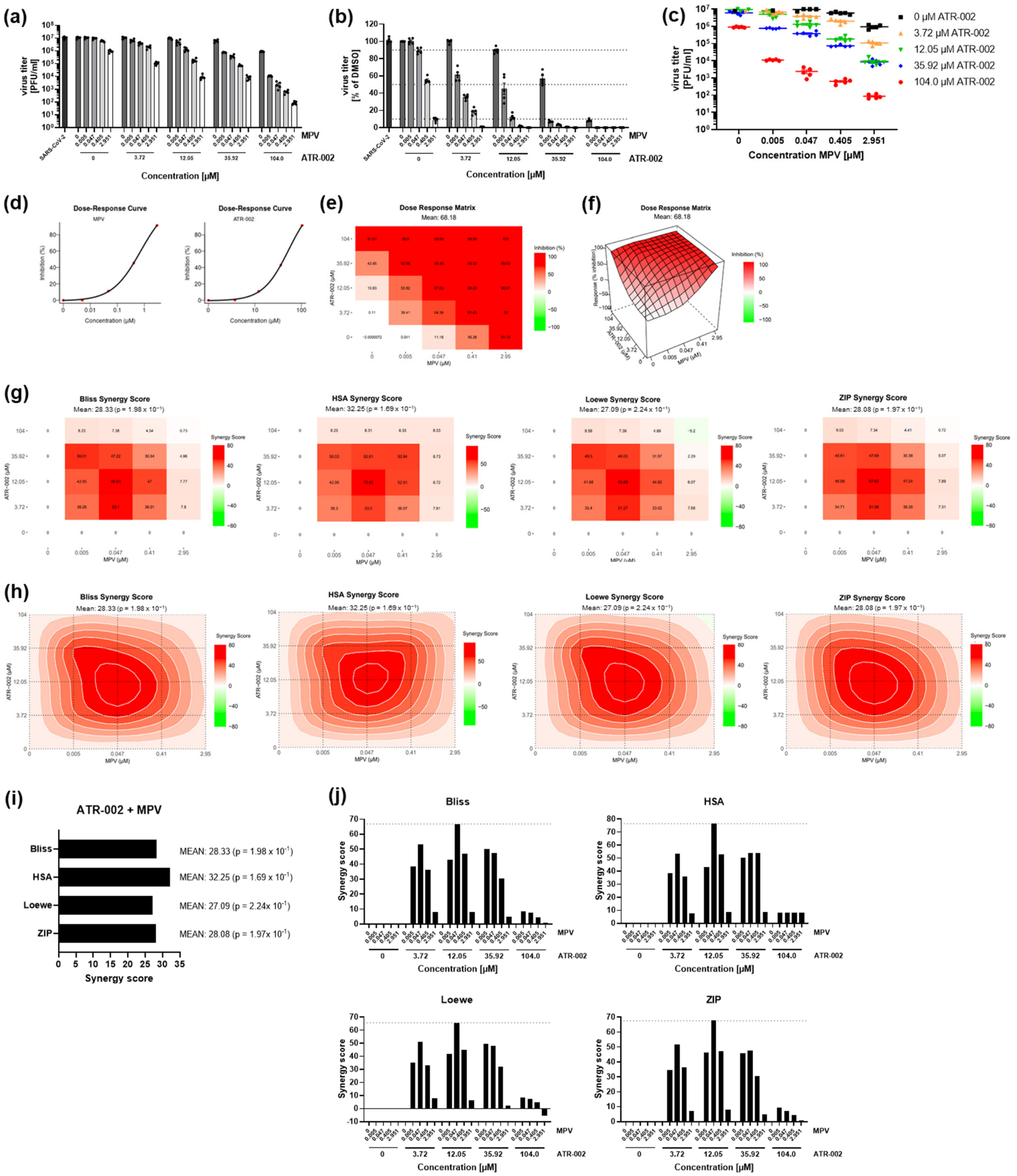
(a)
virus titer [PFU/ml]
SARS-CoV-2
MPV
ATR-002
Concentration [µM]
(b)
virus titer [% of DMSO]
MPV
ATR-002
Concentration [µM]
(c)
virus titer [PFU/ml]
0 µM ATR-002
3.72 µM ATR-002
12.05 µM ATR-002
35.92 µM ATR-002
104.0 µM ATR-002
Concentration MPV [µM]
(d)
Dose-Response Curve
MPV
ATR-002
Inhibition (%)
Concentration (µM)
(e)
Dose Response Matrix
Mean: 68.18
Inhibition (%)
ATR-002 (µM)
MPV (µM)
(f)
Dose Response Matrix
Mean: 68.18
Response (% inhibition)
Inhibition (%)
(g)
Bliss Synergy Score
Mean: 28.33 (p = 1.98 x 10⁻¹)
HSA Synergy Score
Mean: 32.25 (p = 1.69 x 10⁻¹)
Loewe Synergy Score
Mean: 27.09 (p = 2.24 x 10⁻¹)
ZIP Synergy Score
Mean: 28.08 (p = 1.97 x 10⁻¹)
Synergy Score
ATR-002 (µM)
MPV (µM)
(h)
Bliss Synergy Score
HSA Synergy Score
Loewe Synergy Score
ZIP Synergy Score
(i)
ATR-002 + MPV
Bliss
HSA
Loewe
ZIP
MEAN: 28.33 (p = 1.98 x 10⁻¹)
MEAN: 32.25 (p = 1.69 x 10⁻¹)
MEAN: 27.09 (p = 2.24x 10⁻¹)
MEAN: 28.08 (p = 1.97x 10⁻¹)
Synergy score
(j)
Bliss
HSA
Loewe
ZIP
Synergy score
MPV
ATR-002
Concentration [µM]

Figure S3. Determination of the ATR-002 + MPV synergism. ***Related to Figure 3.***

Calu-3 cells were infected with SARS-CoV-2 (MOI: 0.01). 1 h.p.i. drug treatment was initiated. 48 h.p.i. viral titers were determined by plaque titration. (**a**–**c**) Viral titers in PFU/mL (**a**,**c**) or % of DMSO (**b**) of the combinational drug treatments. Data represent means ± SEM of five independent experiments, each performed in triplicates. (**b**) Dashed lines indicate 10 %, 50 % and 90 % titer reduction levels. (**d**) Dose-response curves of the single treatment in combination with DMSO. (**e**) 2D dose response matrix of the ATR-002 + MPV drug combination. (**f**) 3D dose response landscape of the ATR-002 + MPV drug combination. (**g**) 2D heat maps of the 3D landscape visualization and 2D contour shown in Figure 3a and Figure S3h. (**h**) 2D contour visualization of the synergy calculation. (**i**) Synergy score for the ATR-002 + MPV drug combination calculated with the indicated synergy reference models. (**j**) Synergy values of the different drug combinations calculated with the indicated synergy reference models *(see also Table S1)*.

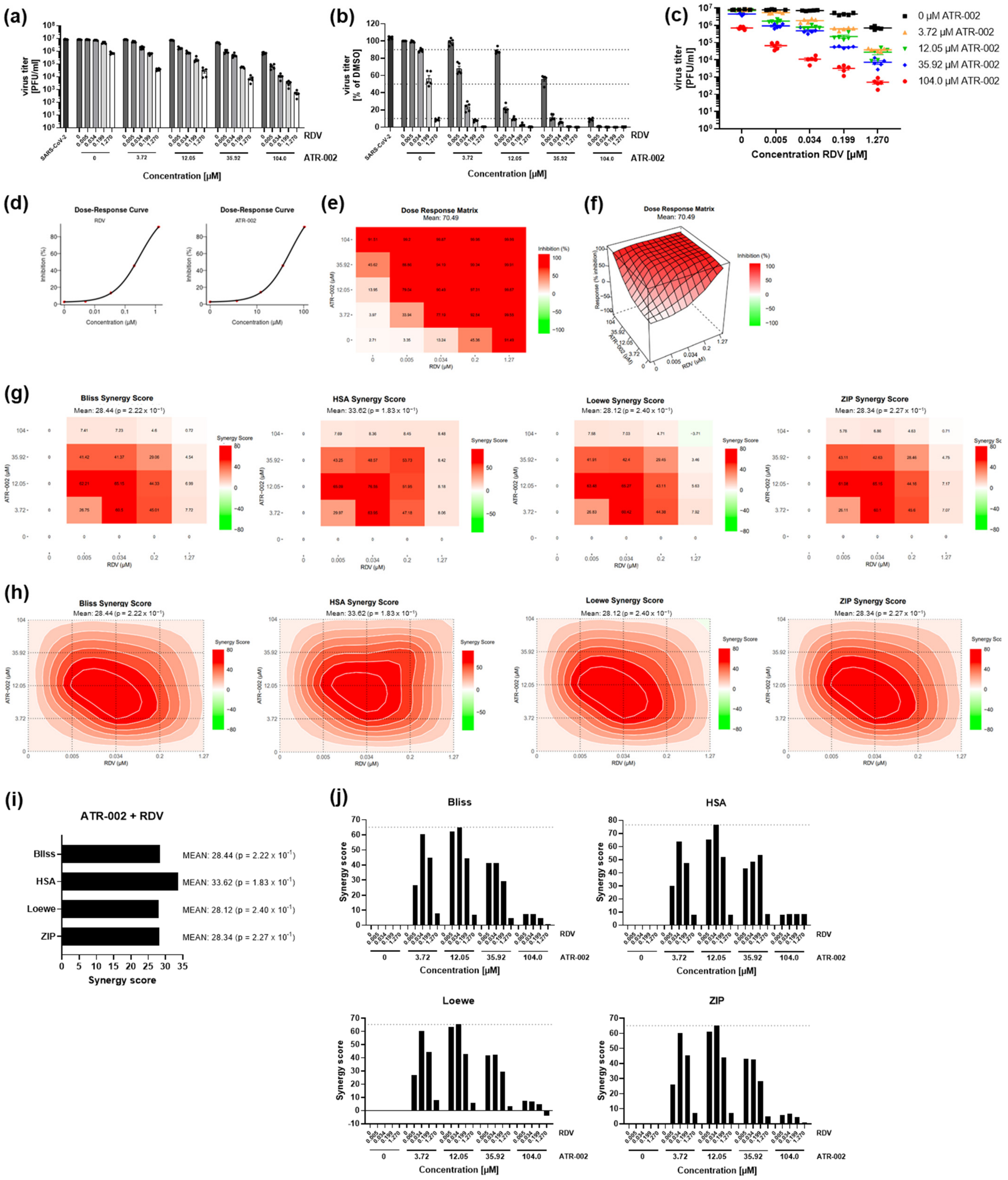
(a)
virus titer [PFU/ml]
RDV
ATR-002
Concentration [µM]
(b)
virus titer [% of DMSO]
RDV
ATR-002
Concentration [µM]
(c)
virus titer [PFU/ml]
0 µM ATR-002
3.72 µM ATR-002
12.05 µM ATR-002
35.92 µM ATR-002
104.0 µM ATR-002
Concentration RDV [µM]
(d)
Dose-Response Curve
(e)
Dose Response Matrix
Mean: 70.49
(f)
Dose Response Matrix
Mean: 70.49
(g)
Bliss Synergy Score
Mean: 28.44 (p = 2.22 x 10⁻¹)
HSA Synergy Score
Mean: 33.62 (p = 1.83 x 10⁻¹)
Loewe Synergy Score
Mean: 28.12 (p = 2.40 x 10⁻¹)
ZIP Synergy Score
Mean: 28.34 (p = 2.27 x 10⁻¹)
(h)
(i)
ATR-002 + RDV
Bliss
HSA
Loewe
ZIP
MEAN: 28.44 (p = 2.22 x 10⁻¹)
MEAN: 33.62 (p = 1.83 x 10⁻¹)
MEAN: 28.12 (p = 2.40 x 10⁻¹)
MEAN: 28.34 (p = 2.27 x 10⁻¹)
Synergy score
(j)
Bliss
HSA
Loewe
ZIP
Synergy score
RDV
ATR-002
Concentration [µM]

Figure S4. Determination of the ATR-002 + RDV synergism. ***Related to Figure 3.***

Calu-3 cells were infected with SARS-CoV-2 (MOI: 0.01). 1 h.p.i. drug treatment was initiated. 48 h.p.i. viral titers were determined by plaque titration. (**a**–**c**) Viral titers in PFU/ml (**a**,**c**) or % of DMSO (b) of the combinational drug treatments. Data represent means ± SEM of five independent experiments, each performed in triplicates. (**b**) Dashed lines indicate 10 %, 50 % and 90 % titer reduction levels. (**d**) Dose-response curves of the single treatment in combination with DMSO. (**e**) 2D dose response matrix of the ATR-002 + RDV drug combination. (**f**) 3D dose response landscape of the ATR-002 + RDV drug combination. (**g**) 2D heat maps of the 3D landscape visualization and 2D contour shown in Figure 3b and Figure S4h. (**h**) 2D contour visualization of the synergy calculation. (**i**) Synergy score for the ATR-002 + RDV drug combination calculated with the indicated synergy reference models. (**j**) Synergy values of the different drug combinations calculated with the indicated synergy reference models *(see also Table S1)*.

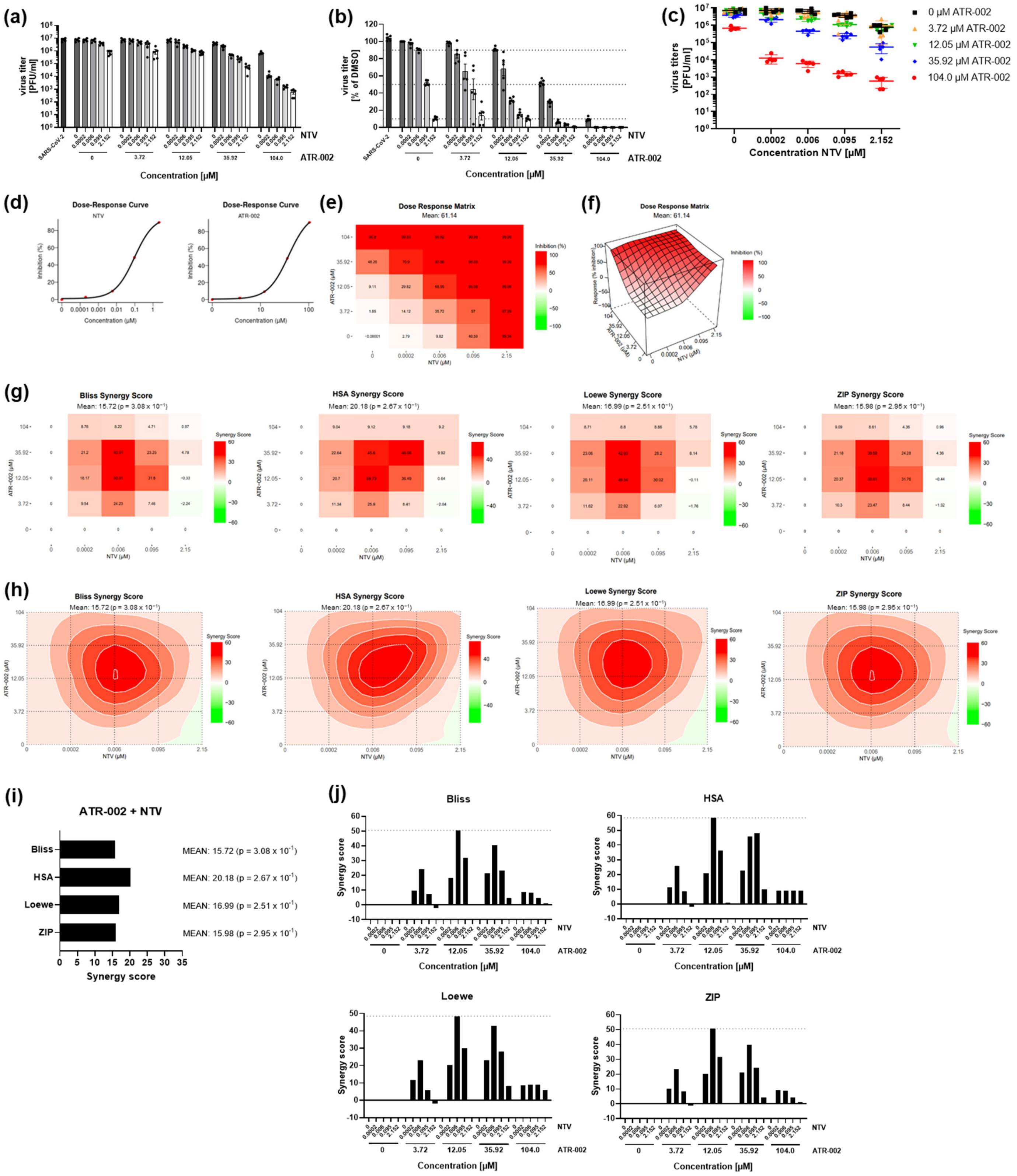
(a)
virus titer [PFU/ml]
NTV
ATR-002
Concentration [μM]
(b)
virus titer [% of DMSO]
NTV
ATR-002
Concentration [μM]
(c)
virus titers [PFU/ml]
Concentration NTV [μM]
0 μM ATR-002
3.72 μM ATR-002
12.05 μM ATR-002
35.92 μM ATR-002
104.0 μM ATR-002
(d)
Dose-Response Curve
NTV
ATR-002
Inhibition (%)
Concentration (μM)
(e)
Dose Response Matrix
Mean: 61.14
Inhibition (%)
ATR-002 (μM)
NTV (μM)
(f)
Dose Response Matrix
Mean: 61.14
Inhibition (%)
(g)
Bliss Synergy Score
Mean: 15.72 (p = 3.08 x 10⁻¹)
HSA Synergy Score
Mean: 20.18 (p = 2.67 x 10⁻¹)
Loewe Synergy Score
Mean: 16.99 (p = 2.51 x 10⁻¹)
ZIP Synergy Score
Mean: 15.98 (p = 2.95 x 10⁻¹)
Synergy Score
(h)
Bliss Synergy Score
HSA Synergy Score
Loewe Synergy Score
ZIP Synergy Score
(i)
ATR-002 + NTV
Bliss
HSA
Loewe
ZIP
MEAN: 15.72 (p = 3.08 x 10⁻¹)
MEAN: 20.18 (p = 2.67 x 10⁻¹)
MEAN: 16.99 (p = 2.51 x 10⁻¹)
MEAN: 15.98 (p = 2.95 x 10⁻¹)
Synergy score
(j)
Bliss
HSA
Loewe
ZIP
Synergy score
NTV
ATR-002
Concentration [μM]

Figure S5. Determination of the ATR-002 + NTV synergism. ***Related to Figure 3.***

Calu-3 cells were infected with SARS-CoV-2 (MOI: 0.01). 1 h.p.i. drug treatment was initiated. 48 h.p.i. viral titers were determined by plaque titration. (**a**–**c**) Viral titers in PFU/mL (**a**,**c**) or % of DMSO (**b**) of the combinational drug treatments. Data represent means ± SEM of five independent experiments, each performed in triplicates. (**b**) Dashed lines indicate 10 %, 50 % and 90 % titer reduction levels. (**d**) Dose-response curves of the single treatment in combination with DMSO. (**e**) 2D dose response matrix of the ATR-002 + NTV drug combination. (**f**) 3D dose response landscape of the ATR-002 + NTV drug combination. (**g**) 2D heat maps of the 3D landscape visualization and 2D contour shown in Figure 3c and Figure S5h. (**h**) 2D contour visualization of the synergy calculation. (**i**) Synergy score for the ATR-002 + NTV drug combination calculated with the indicated synergy reference models. (**j**) Synergy values of the different drug combinations calculated with the indicated synergy reference models *(see also Table S1)*.

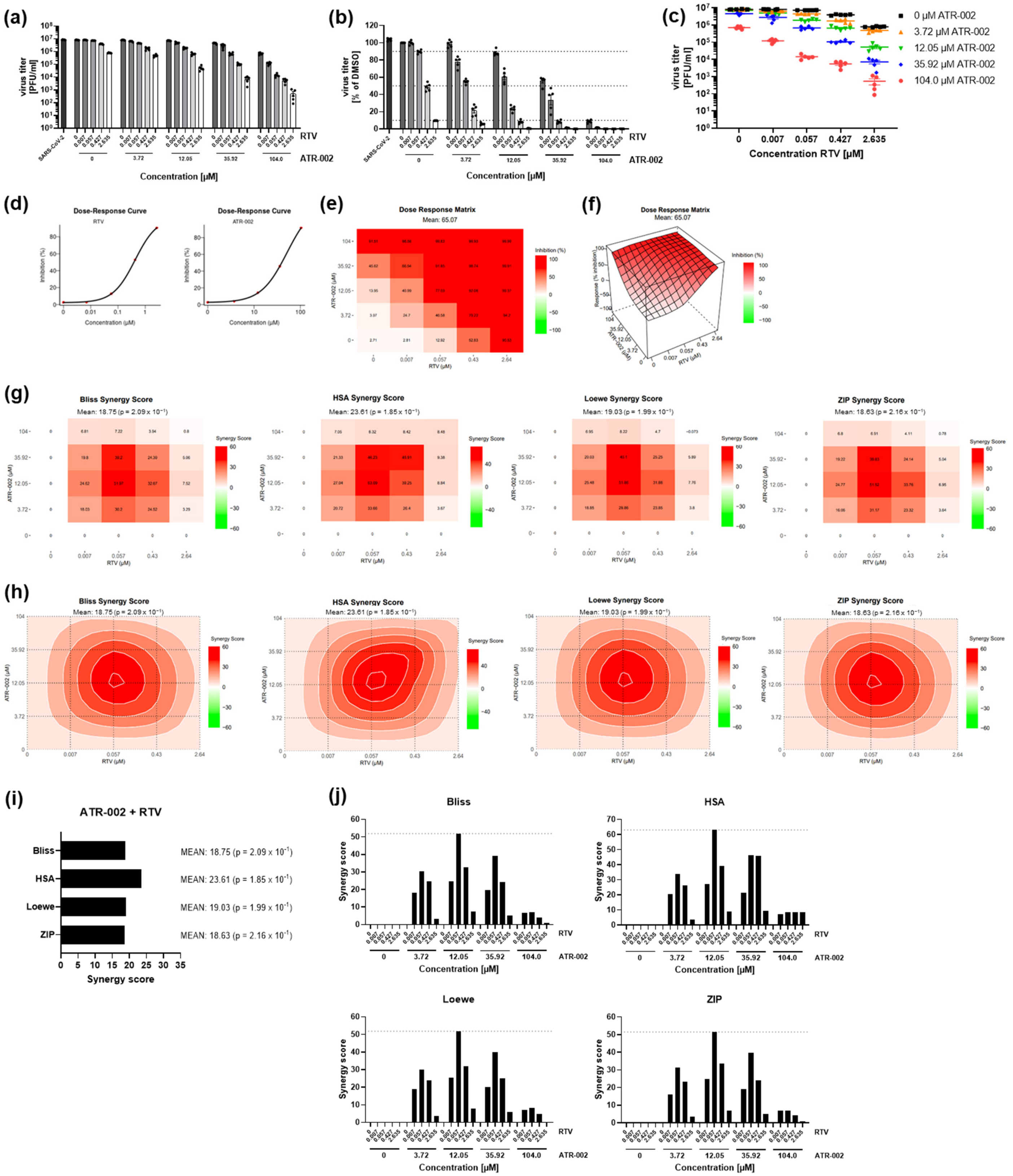

(a)
virus titer [PFU/ml]
SARS-CoV-2
RTV
ATR-002
Concentration [µM]
(b)
virus titer [% of DMSO]
SARS-CoV-2
RTV
ATR-002
Concentration [µM]
(c)
virus titer [PFU/ml]
0 µM ATR-002
3.72 µM ATR-002
12.05 µM ATR-002
35.92 µM ATR-002
104.0 µM ATR-002
Concentration RTV [µM]
(d)
Dose-Response Curve
RTV
Inhibition (%)
Concentration (µM)
Dose-Response Curve
ATR-002
Inhibition (%)
Concentration (µM)
(e)
Dose Response Matrix
Mean: 65.07
ATR-002 (µM)
RTV (µM)
Inhibition (%)
(f)
Dose Response Matrix
Mean: 65.07
Response (% inhibition)
ATR-002 (µM)
RTV (µM)
Inhibition (%)
(g)
Bliss Synergy Score
Mean: 18.75 (p = 2.09 x 10⁻¹)
HSA Synergy Score
Mean: 23.61 (p = 1.85 x 10⁻¹)
Loewe Synergy Score
Mean: 19.03 (p = 1.99 x 10⁻¹)
ZIP Synergy Score
Mean: 18.63 (p = 2.16 x 10⁻¹)
Synergy Score
ATR-002 (µM)
RTV (µM)
(h)
Bliss Synergy Score
Mean: 18.75 (p = 2.09 x 10⁻¹)
HSA Synergy Score
Mean: 23.61 (p = 1.85 x 10⁻¹)
Loewe Synergy Score
Mean: 19.03 (p = 1.99 x 10⁻¹)
ZIP Synergy Score
Mean: 18.63 (p = 2.16 x 10⁻¹)
Synergy Score
ATR-002 (µM)
RTV (µM)
(i)
ATR-002 + RTV
Bliss
HSA
Loewe
ZIP
MEAN: 18.75 (p = 2.09 x 10⁻¹)
MEAN: 23.61 (p = 1.85 x 10⁻¹)
MEAN: 19.03 (p = 1.99 x 10⁻¹)
MEAN: 18.63 (p = 2.16 x 10⁻¹)
Synergy score
(j)
Bliss
HSA
Loewe
ZIP
Synergy score
RTV
ATR-002
Concentration [µM]

Figure S6. Determination of the ATR-002 + RTV synergism. ***Related to Figure 3.***

Calu-3 cells were infected with SARS-CoV-2 (MOI: 0.01). 1 h.p.i. drug treatment was initiated. 48 h.p.i. viral titers were determined by plaque titration. (**a**–**c**) Viral titers in PFU/mL (**a**,**c**) or % of DMSO (**b**) of the combinational drug treatments. Data represent means ± SEM of five independent experiments, each performed in triplicates. (**b**) Dashed lines indicate 10 %, 50 % and 90 % titer reduction levels. (**d**) Dose-response curves of the single treatment in combination with DMSO. (**e**) 2D dose response matrix of the ATR-002 + RTV drug combination. (**f**) 3D dose response landscape of the ATR-002 + RTV drug combination. (**g**) 2D heat maps of the 3D landscape visualization and 2D contour shown in Figure 3d and Figure S6h. (**h**) 2D contour visualization of the synergy calculation. (**i**) Synergy score for the ATR-002 + RTV drug combination calculated with the indicated synergy reference models. (**j**) Synergy values of the different drug combinations calculated with the indicated synergy reference models *(see also Table S1)*.

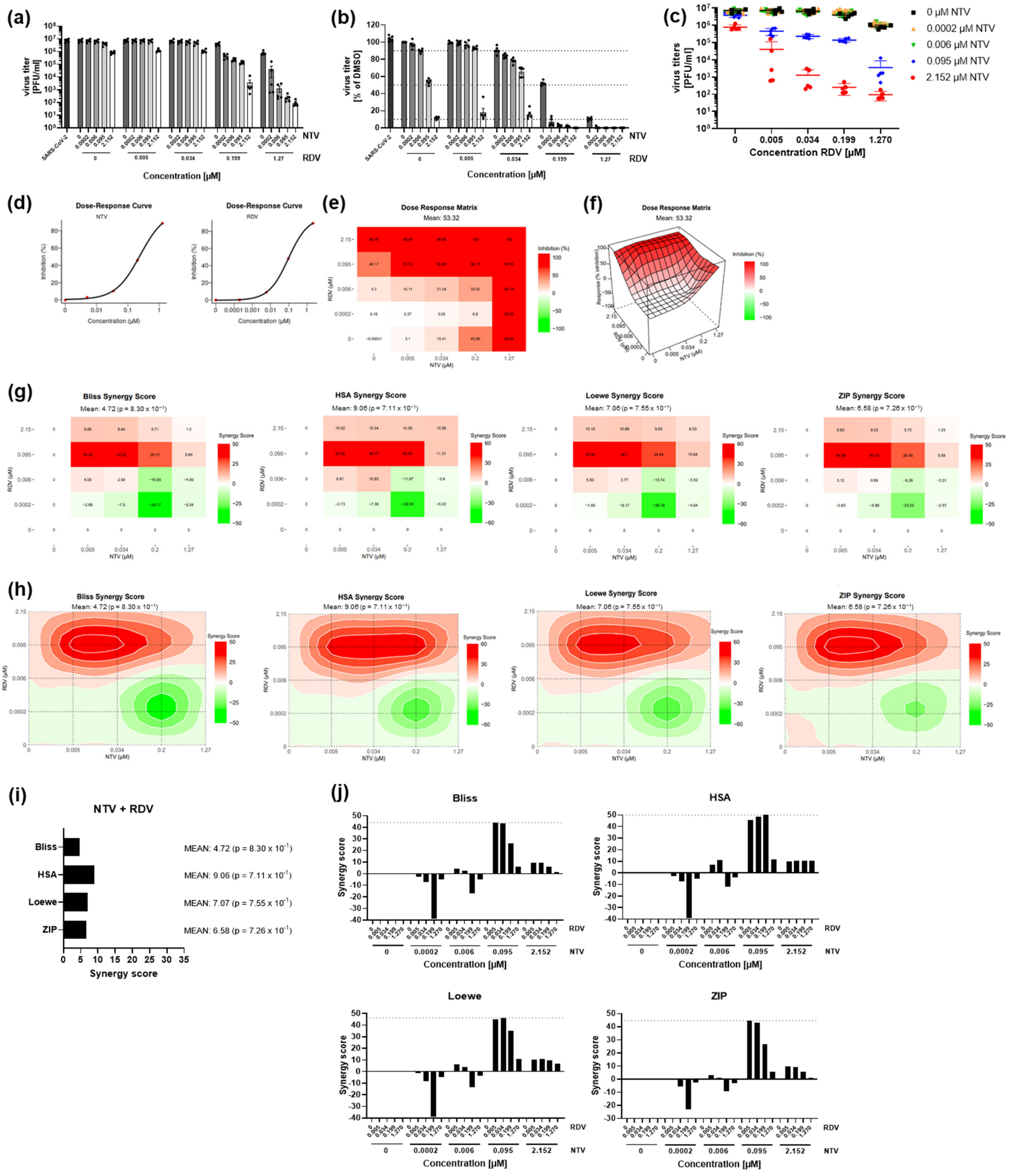
(a)
virus titer [PFU/ml]
NTV
RDV
Concentration [µM]
(b)
virus titer [% of DMSO]
Concentration [µM]
(c)
virus titers [PFU/ml]
Concentration RDV [µM]
0 µM NTV
0.0002 µM NTV
0.006 µM NTV
0.095 µM NTV
2.152 µM NTV
(d)
Dose-Response Curve
Concentration (µM)
(e)
Dose Response Matrix
Mean: 53.32
(f)
Dose Response Matrix
Mean: 53.32
(g)
Bliss Synergy Score
Mean: 4.72 (p = 8.30 x 10⁻¹)
HSA Synergy Score
Mean: 9.06 (p = 7.11 x 10⁻¹)
Loewe Synergy Score
Mean: 7.06 (p = 7.55 x 10⁻¹)
ZIP Synergy Score
Mean: 6.58 (p = 7.26 x 10⁻¹)
(h)
(i)
NTV + RDV
Bliss
HSA
Loewe
ZIP
MEAN: 4.72 (p = 8.30 x 10-1)
MEAN: 9.06 (p = 7.11 x 10-1)
MEAN: 7.07 (p = 7.55 x 10-1)
MEAN: 6.58 (p = 7.26 x 10-1)
Synergy score
(j)
Bliss
HSA
Loewe
ZIP
Synergy score
Concentration [µM]

Figure S7. Determination of the RDV + NTV synergism. ***Related to Figure 3.***

Calu-3 cells were infected with SARS-CoV-2 (MOI: 0.01). 1 h.p.i. drug treatment was initiated. 48 h.p.i. viral titers were determined by plaque titration. (**a**–**c**) Viral titers in PFU/mL (**a**,**c**) or % of DMSO (**b**) of the combinational drug treatments. Data represent means ± SEM of five independent experiments, each performed in triplicates. (**b**) Dashed lines indicate 10 %, 50 % and 90 % titer reduction levels. (**d**) Dose-response curves of the single treatment in combination with DMSO. (**e**) 2D dose response matrix of the RDV + NTV drug combination. (**f**) 3D dose response landscape of the RDV + NTV drug combination. (**g**) 2D heat maps of the 3D landscape visualization and 2D contour shown in Figure 3e and Figure S7h. (**h**) 2D contour visualization of the synergy calculation. (**i**) Synergy score for the RDV + NTV drug combination calculated with the indicated synergy reference models. (**j**) Synergy values of the different drug combinations calculated with the indicated synergy reference models *(see also Table S1)*.

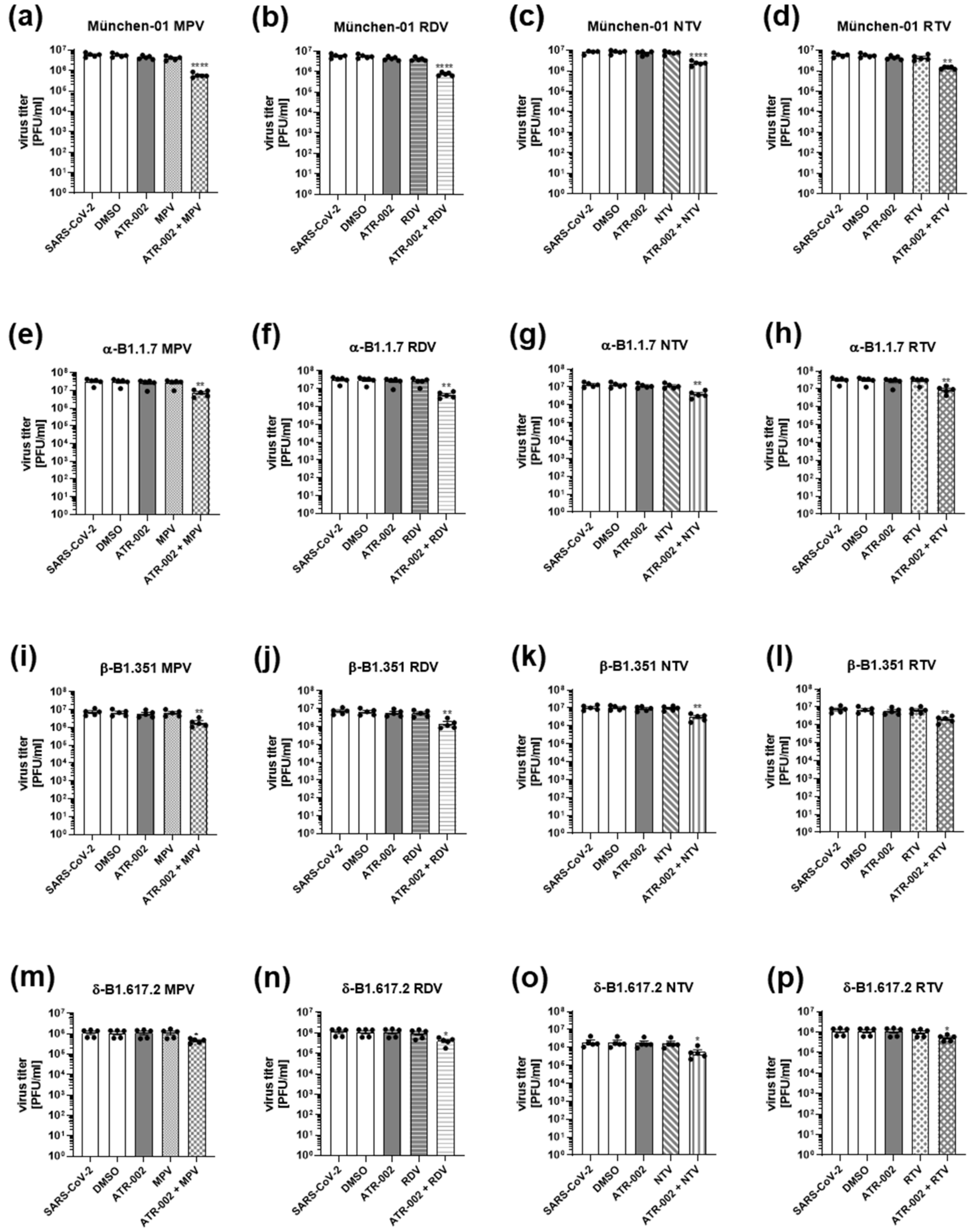

Figure S8. Synergistic effects of different drug combinations on the replication of SARS-CoV-2 variants. ***Related to Figure 4.***

Calu-3 cells were infected with SARS-CoV-2 (MOI: 0.01). 1 h.p.i. drug treatment was initiated. MPV (0.047 µM) (**a**,**e**,**i**,**m**), RDV (0.034 µM) (**b**,**f**,**j**,**n**), NTV (0.006 µM) (**c**,**g**,**k**,**o**) or RTV (0.057 µM) (**d**,**h**,**l**,**p**) were combined with ATR-002 (12.05 µM). Untreated (SARS-CoV-2), DMSO treated and single treated (drug + DMSO) cells served as controls. 48 h.p.i. viral titers were determined by plaque titration. Shown are means ± SEM five independent experiments, each performed in triplicates. Significance was calculated using an unpaired *t*-test for each inhibitor individually, comparing the single treated (MPV, RDV, NTV, RTV) vs. the double treated results (* $p \leq 0.0332$; ** $p \leq 0.0021$; **** $p \leq 0.0001$).

Table S1. Highest synergy score values.

Highest synergy score values, calculated for the drug combinations MPV 0.047 µM / ATR-002 12.05 µM, RDV 0.034 µM / ATR-002 12.05 µM, NTV 0.006 µM / ATR-002 12.05 µM, RTV 0.057 µM / ATR-002 12.05 µM and RDV / NTV (Bliss, Loewe, ZIP: RDV 0.034 µM + NTV 0.095 µM; HSA: RDV 0.199 µM + NTV 0.095 µM) *Related to Figure S3–S7j.*

Drug combination	Reference model	Synergy score
MPV / ATR-002	Bliss	66.83
	HSA	76.45
	Loewe	65.69
	ZIP	67.63
RDV / ATR-002	Bliss	65.15
	HSA	76.55
	Loewe	65.27
	ZIP	65.15
NTV / ATR-002	Bliss	50.51
	HSA	58.73
	Loewe	48.54
	ZIP	50.61
RTV / ATR-002	Bliss	51.97
	HSA	63.09
	Loewe	51.86
	ZIP	51.52
RDV / NTV	Bliss	44.01
	HSA	49.95
	Loewe	46.10
	ZIP	44.59

Table S2. Drug combination sensitivity score (CSS).

	ATR-002 + MPV	ATR-002 + RDV	ATR-002 + NTV	ATR-002 + RTV	NTV + RDV
CSS	98.25	98.12	90.01	94.63	80.1
